# A Novel Method for Ocean Wind Speed Detection Based on Energy Distribution of Beidou Reflections

**DOI:** 10.3390/s19122779

**Published:** 2019-06-20

**Authors:** Qiang Wang, Yunlong Zhu, Kittipong Kasantikul

**Affiliations:** School of Electronic and Information Engineering, Beihang University (BUAA), Beijing 100191, China; wangqiang_620@163.com (Q.W.); kittipong.mut@gmail.com (K.K.)

**Keywords:** GNSS-R, wind speed, energy zone, Beidou

## Abstract

The Global Navigation Satellite System Reflectometry (GNSS-R) technique exploits the characteristics of reflected GNSS signals to estimate the geophysical parameters of the earth’s surface. This paper focuses on investigating the wind speed retrieval method using ocean scattered signals from a Beidou Geostationary Earth Orbit (GEO) satellite. Two new observables are proposed by computing the ratio of the low energy zone and the high energy zone of the delay waveform. Coastal experimental raw data from a Beidou GEO satellite are processed to establish the relationship between the energy-related observables and the sea surface wind. When the delay waveform normalized amplitude (this will be referred to as “threshold” in what follows) is 0.3, fitting results show that the coefficient of determination is more than 0.76 in the gentle wind scenario (<10 m/s), with a root mean square error (RMSE) of less than 1.0 m/s. In the Typhoon UTOR scenario (12.7 m/s~37.3 m/s), the correlation level exceeds 0.82 when the threshold is 0.25, with a RMSE of less than 3.10 m/s. Finally, the impact of the threshold and coherent integration time on wind speed retrieval is discussed to obtain an optimal result. When the coherent integration time is 50 milliseconds and the threshold is 0.15, the best wind speed retrieval error of 2.63 m/s and a correlation level of 0.871 are obtained in the UTOR scenario.

## 1. Introduction

As one of the most important links in the global climate system, the ocean plays a decisive role in regulating the climate through the exchange of energy with the atmosphere and water circulation. Coastal areas are threatened by storms and typhoons, especially in the northwest Pacific Ocean. Therefore, monitoring the offshore sea-state is necessary to ensure the safety of social activities in local areas. Traditional methods such as buoys and active radars have performed well in sea-state detection. However, their high cost and geographic dependence limits their quantitative distribution. The Global Navigation Satellite System Reflectometry (GNSS-R) technique has been an innovative option for remote sensing since it was first proposed for mesoscale altimetry by Martin-Neira in 1993 [1]. This technique exploits signals of opportunity from GPS or other GNSS constellations (Galileo, Beidou, Glonass, etc.) being reflected off the Earth’s surface to retrieve various geophysical parameters of the Earth’s surface. During the initial period, scientists mainly focused on GNSS-R based ocean remote sensing, such as sea altimetry and scattermetry [1,2,3,4,5,6,7,8]. During the last two decades, the applications of the GNSS-R technique have expanded to various fields, such as monitoring of sea-ice, sea salinity, snow depth, oil spilling and soil moisture [9,10,11,12,13]. Meanwhile, various experimental activities have been performed to demonstrate the performance of this technique. A detailed review of GNSS-R principles, applications and future space-borne missions can be found in [14,15].

Among others, sea surface wind speed measurement has been one of the most important applications of the GNSS-R technique. Through processing data from an airborne experiment, ocean wind measurement based on GPS reflections was first presented in [5]. Since then, several algorithms and observables have been proposed to retrieve sea surface wind speed and sea state in different experimental scenarios, including ground-borne, air-borne and space-borne [16,17,18,19,20,21,22]. Among them, some observables are proposed to develop empirical wind retrieval models, which are defined as the descriptors of “size” or “shape” of 1-D delay waveforms or 2-D delay-Doppler maps (DDM) such as DDM area and volume within a given threshold [18,23,24]. These observables perform well for wind speed retrieval both in airborne and space-borne experiment scenarios. Nevertheless, these observables do not perform well in low altitude experience scenarios due to the limited extension of the glistening zone [14]. By considering the coherent and incoherent component of the received signal, some other observables such as coherent time and effectiveness of incoherent averaging are proposed and related to the significant wave height (SWH) [6,20,25]. When the specular component is dominant in the scattering process, these observables perform well in low altitude platform, especially in ground-based scenarios. In [26], the delay-related and spectral-related observables, defined as relative-amplitude and Doppler centroid, respectively, are proposed for typhoon wind retrieval. In [27], the ratio between coherent and incoherent components was discussed for coastal wind retrieval.

Beidou consists of a hybrid constellation with the satellites in different orbits, including the inclined geosynchronous satellite orbit (IGSO), the geostationary earth orbit (GEO) and the medium earth orbit (MEO). Until now, with 22 satellites in-orbit, Beidou has provided integrated position, velocity and time (PVT) services in Asia–Pacific regions. Therefore, a mass of reflections over the sea surface from Beidou satellites can be used for remote sensing. In [28], an exponential empirical model for monitoring wind speed was established based on the coherent time of Beidou reflections. In addition, researchers also demonstrated the feasibility of ocean altimetry, employing reflected Beidou signals in [29]. 

This paper proposes two new observables for estimating wind speed by analyzing the complex waveform, which describes the variation of energy distribution of reflected signals. In Section 2, the model of GNSS-R waveform is reviewed and the discussion on the energy distribution of complex waveform is presented through simulation. Two new observables related to energy distribution of complex waveform are proposed to retrieve wind speed. In Section 3, this paper summarizes the main scenarios of the coastal experiment in the Yangjiang site and the raw data preprocessing chain from the Beidou GEO satellite. Then, the analysis of the results is presented in Section 4. In addition, the influences of the threshold and coherent integration time on wind speed retrieval are discussed to obtain an optimal retrieval result. Finally, the conclusions and future work are presented.

## 2. Theoretical Analysis and Methods 

### 2.1. Theoretical Analysis

This section presents the analysis of the relationship between GNSS-R waveforms and wind speed to derive the sensitivity of the observable. The shape of GNSS-R waveform can characterize the sea surface roughness: the higher the wind speed is, the rougher the sea surface is and, thus, the extension of the waveform is. This section focuses on the description of the energy distribution of the GNSS-R waveform and defines the bistatic radar model, which describes the total average correlation power of the scattered GNSS signal as a function of the time delay Δτ and the frequency offset Δf with respect to the time delay and Doppler frequency associate with the nominal specular point on the ocean’s surface [30].

(1) $$\begin{array}{ll} \langle Y_{r}\left(\Delta \tau ,\Delta f\right)\rangle \propto & \iint_{D}\frac{G_{r}^{2}\left(\rho \right)}{R_{ts}^{2}\left(\rho \right)\cdot R_{sr}^{2}\left(\rho \right)}\cdot R_{AC}^{2}\left(\Delta \tau -\tau \left(\rho \right)\right)\\ & \;\cdot {\left|S\left(\Delta f-f\left(\rho \right)\right)\right|}^{2}\cdot \sigma^{0}d\rho \end{array}$$

where $\langle Y_{r}\left(\cdot \right)\rangle$ is the averaged power waveform; D is the integration area; $G_{r}$ is the receiver’s antenna gain, $R_{ts}$ is the distance from the transmitter to the reflection point ρ; $R_{sr}$ is the distance from ρ to the receiver. Δτ and Δf are delay offset and Doppler frequency offset, respectively. τ(ρ) and f(ρ) are the delay offset and Doppler frequency shift at the scatter point, respectively. $R_{AC}$ is the auto-correlation function of the GNSS ranging code defined as $R_{AC}\left(\tau \right)=1-\tau /\tau_{c}$ when $\left|\tau \right|\leq \tau_{c}$, $R_{AC}\left(\tau \right)=0$, elsewhere ($\tau_{c}$ is the length of one chip of the C/A code). |S| is the Doppler frequency filter function defined as |S|=|sin(πf)/(πf)|. $\sigma^{0}$ is the normalized bi-static radar cross section of the sea surface, which is calculated and expressed as a function of the probability density function (PDF) of the slopes based on the geometric optics limit.

(2) $$\sigma^{0}=\pi {\left|ℜ\right|}^{2}{\left(\frac{q}{q_{z}}\right)}^{4}P\left(\frac{-q_{⊥}}{q_{z}}\right)$$

where ℜ is the Fresnel reflection coefficient, q is the scattering vector, P is the PDF of the surface slope.

In the following simulation, the PDF is assumed to be a 2-D zero-mean Gaussian distribution [31]. The mean square slopes of the up-wind and cross-wind are approximated based on the simplified sea roughness model proposed by Katzberg [32] as

(3) $$\begin{array}{l} \sigma_{u}=0.45\cdot \left[0.000+0.00316\cdot f\left(u\right)\right]\\ \sigma_{c}=0.45\cdot \left[0.003+0.00192\cdot f\left(u\right)\right] \end{array}$$

where $\sigma_{u}$ and $\sigma_{c}$ are the mean square slopes of the up-wind and cross-wind, respectively. f(u) is the function of the wind speed at the height of 10 m above the sea surface and is calculated as

(4) $$f\left(u\right)=\left\{ \begin{array}{l} u,\;\;\;\;\;0<u<3.49\\ 6\ln\left(u\right)-4.0,\;3.49<u<46\\ 0.441u,\;\;\;\;46<u \end{array}\right..$$

To analyze the relationship between the wind speed and energy distribution of the waveform, a simulation is performed with the parameters listed in Table 1.

**Figure 1:**
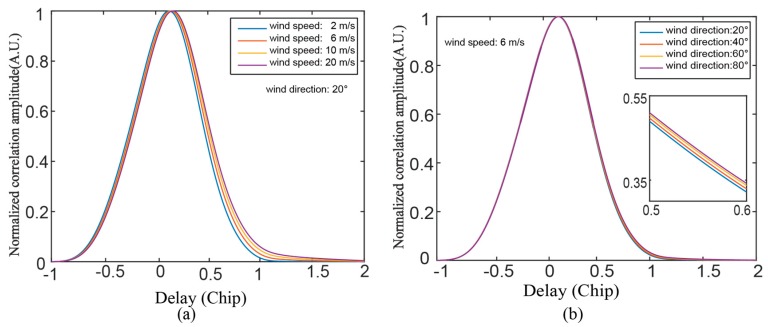
Simulation of the 1-D delay waveform for different wind conditions: (**a**) Wind speed from 2 m/s to 20 m/s, wind direction: 20°. (**b**) Wind directions from 20° to 80°, wind speed: 6 m/s.

Considering the rather small effect of the Doppler frequency spreading in the following experimental scenario, the simulation mainly focuses on the variation of the delay waveform. Figure 1 shows the normalized delay waveforms under different wind conditions. As shown in Figure 1a, we set a constant wind direction of 20 deg and simulate 1-D delay waveforms corresponding to different wind speeds. The extension of the trailing edge shows a dependence on wind speed, while the leading edge of the waveform remains relatively stable with different wind speeds. As shown in Figure 1b, we set a constant wind speed of 6 m/s and simulate the waveforms corresponding to different wind directions. This shows a slight change in the trailing edge of the waveform. 

### 2.2. Methods

**Figure 2:**
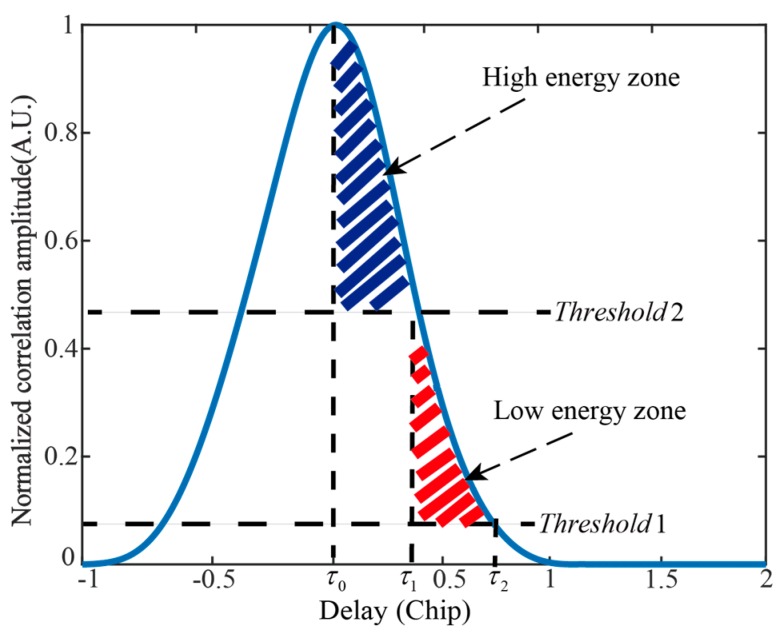
Definition of the low energy zone and the high energy zone from the normalized delay waveform.

The trailing edge of the delay waveform is divided into two parts, named the low energy zone and the high energy zone, respectively. Figure 2 illustrates these two energy zones at a wind speed of 2 m/s.

As shown in Figure 2, the trailing edge is divided into two energy zones by two thresholds. We set the $Threshold1=P_{noise}$, where $P_{noise}$ is the noise power. Threshold2 is the boundary value between the high energy zone and low energy zone: Threshold1<Threshold2<1. A detailed discussion on threshold is presented in Section 4.3. The delay range of the high energy zone is $\left(\tau_{0},\tau_{1}\right)$, $\tau_{0}$ and $\tau_{1}$ are the delay values when the correlation amplitudes equal maximum and Threshold2, respectively. And the delay range of the low energy zone is $\left(\tau_{1},\tau_{2}\right)$, where $\tau_{2}$ is the delay value when the amplitude equals Threshold1. The description of the new observables that are used to derive wind speed is provided in the following section.

#### 2.2.1. Averaged Amplitude Ratio of Low Energy Zone and High Energy Zone

The first observable named EMR is the averaged correlation amplitudes ratio of the low energy zone and high energy zone. And the EMR is shown as

(5) $$EMR=\frac{{\bar{Y}}_{r}^{l}}{{\bar{Y}}_{r}^{h}}$$

where ${\bar{Y}}_{r}^{l}$ and ${\bar{Y}}_{r}^{h}$ represent the average amplitudes of the low energy zone and high energy zone

(6) $$\left\{ \begin{array}{l} {\bar{Y}}_{r}^{l}=\frac{1}{\tau_{2}-\tau_{1}}\int_{\tau_{1}}^{\tau_{2}}Y_{r}\left(\tau \right)d\tau \\ {\bar{Y}}_{r}^{h}=\frac{1}{\tau_{1}-\tau_{0}}\int_{\tau_{0}}^{\tau_{1}}Y_{r}\left(\tau \right)d\tau \end{array}\right.$$

where $\tau_{1}$ and $\tau_{2}$ are the delay values when the correlation amplitudes equal Thershold2 and Thershold1, respectively. $\tau_{0}$ is the delay value when the amplitude is the maximum.

#### 2.2.2. Area Ratio of the Low Energy Zone and High Energy Zone

The second observable named EDR is the area ratio of the low energy zone and high energy zone

(7) $$EDR=\frac{Area_{l}}{Area_{h}}$$

where $Area_{l}$ and $Area_{h}$ are the areas of low energy zone and high energy zone, respectively.

(8) $$\left\{ \begin{array}{l} Area_{l}=\int_{\tau_{1}}^{\tau_{2}}Y_{r}\left(\tau \right)d\tau \\ Area_{h}=\int_{\tau_{0}}^{\tau_{1}}Y_{r}\left(\tau \right)d\tau \end{array}\right.$$

## 3. Coastal Experiment Description

### 3.1. Experimental Setup

The coastal experiment was conducted to observe typhoon events in the Yangjiang site during the summer of 2013 within the cooperation of the ESA-China in GNSS Reflectometry [25].

**Figure 3:**
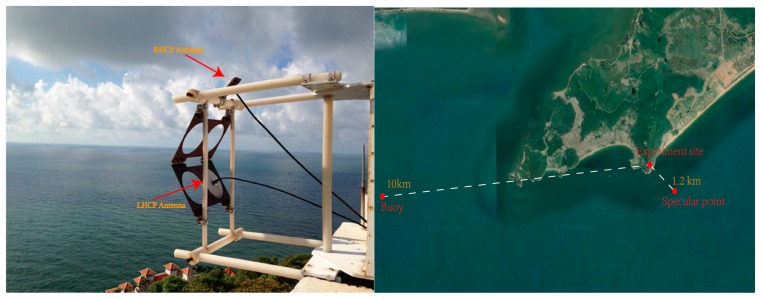
Scenario and experiment setup in the Yangjiang site.

As shown in Figure 3, the antennas were mounted directly on the roof of the building in the mountain (21.56°N, 111.86°E), with approximately 120 m altitude above the sea’s surface. Both antennas are compatible with frequencies of 1575.42 MHz and 1561.098 MHz. The right-hand circular polarization antenna was used to collect direct signals from GNSS satellites with zenith-looking and the left-hand circular polarization antenna pointing toward the sea surface was used to collect reflected signals, as shown in Figure 3. To collect the weak reflected signal, the left-handed circular one has a high antenna gain of 12 dB and a narrow beam width of 38°. With a detailed parameter shown in Table 2, a two channels GNSS receiver was employed to collect GPS and Beidou signals.

During the experiment, Beidou signals were collected with a fixed time length of 250 s each time. In this paper, the reflected signals from the Beidou GEO4 satellite (with the elevation of 31° and azimuth of 108°) are processed. The in situ wind speed measurements from the Zhapo meteorological station (No. 59674) are used to assess the performances of the results.

### 3.2. Data Processing

The data processing mainly included three parts: raw data preprocessing, averaged-waveform computation and observable computation.

• Raw data preprocessing.

**Figure 4:**
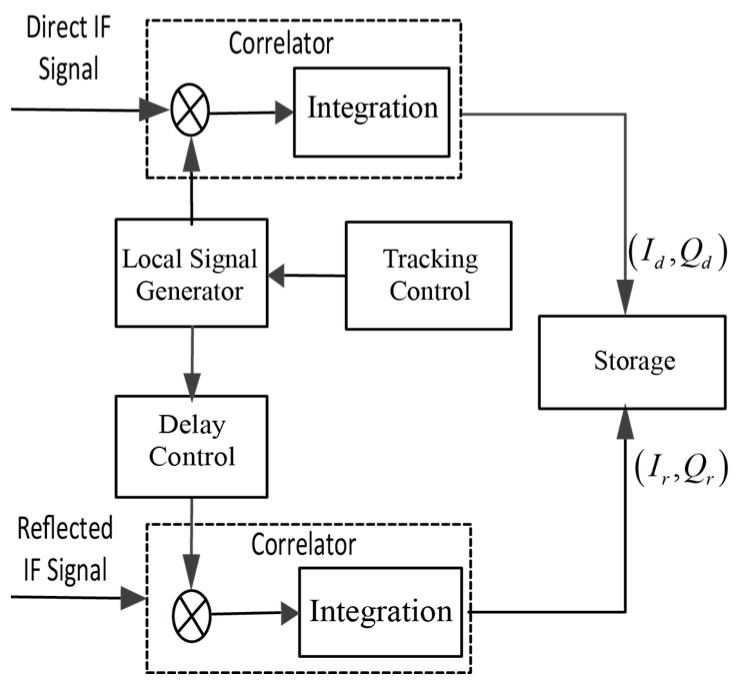
Simplified block diagram of data preprocessing.

The Direct intermediate frequency (IF) signals were tracked to calculate the precise code delay and the Doppler frequency. The reflected IF signals were cross-correlated against local generate code replicas with different delays which were estimated by using the direct signal code phase as shown in Figure 4.

The output 1 ms complex waveform of the reflected signal is

(9) $$y_{r}\left(t,\tau \right)=I_{r}\left(t,\tau \right)+j\cdot Q_{r}\left(t,\tau \right)$$

where $I_{r}\left(t,\tau \right)$ and $Q_{r}\left(t,\tau \right)$ are the in-phase component and quadrature component of the complex waveform, respectively.

• Averaged-waveform computation

**Figure 5:**
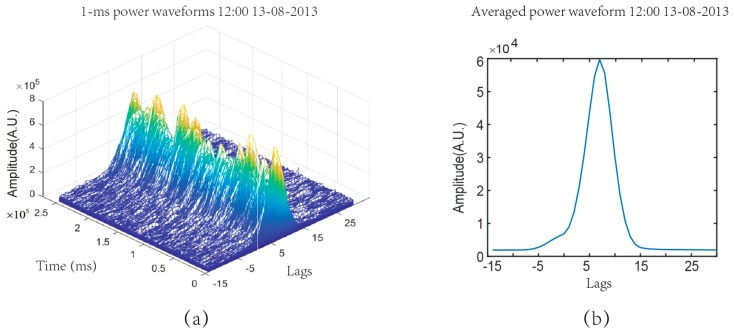
Power waveforms at 12:00 on August 13, 2013. (**a**) 1 ms power waveforms; (**b**) Averaged power waveform (coherent integration time = 50 ms, incoherent integration: *N* = 5000).

In the experimental scenario, the collected reflected signals were contaminated by different kinds of factors, such as thermal noise, speckle noise and direct signal crosstalk. It is necessary to post-process the waveform from the Beidou-Reflected software receiver. As discussed in [33], the coherent and incoherent averaging were employed to increase the SNR of the waveform. Here, the coherent integration time was set to 50 ms. Then, utilizing the difference coherence properties between direct and reflected signals, the cross-talk of direct signals was removed by

(10) $$Y_{r}\left(\tau \right)\;=\;\frac{1}{N}{\sum_{k=1}^{N}\left|y_{r\_50}\left(t_{k},\tau \right)\;-\;\frac{1}{N}\sum_{k=1}^{N}y_{r\_50}\left(t_{k},\tau \right)\right|}^{2}$$

where $Y_{r}\left(\tau \right)$ is the averaged power waveform. $y_{r\_50}\left(t_{k},\tau \right)$ is the 50 ms coherent integrated complex waveform, N is the number of incoherent averages (*N* = 5000). Figure 5 shows the power waveforms at 12:00 on August 13, 2013 in the Yangjiang experiment. Figure 5a plots 250,000 consecutive 1 ms power waveforms. Figure 5b plots the averaged power waveform.

• Observable computation

The new proposed observables EMR and EDR were extracted from the normalized power waveform during the Yangjiang coastal experiment, respectively.

## 4. Results, Analysis and Discussion

To investigate the performance of the proposed method, we processed the reflected signals from the Beidou GEO4 satellite from August 3 to August 5 and from August 13 to August 14 in 2013. In the second period, the Typhoon UTOR approached the coast of Yangjiang of Guangdong province.

### 4.1. Wind Speed from the Meteorological Station

**Figure 6:**
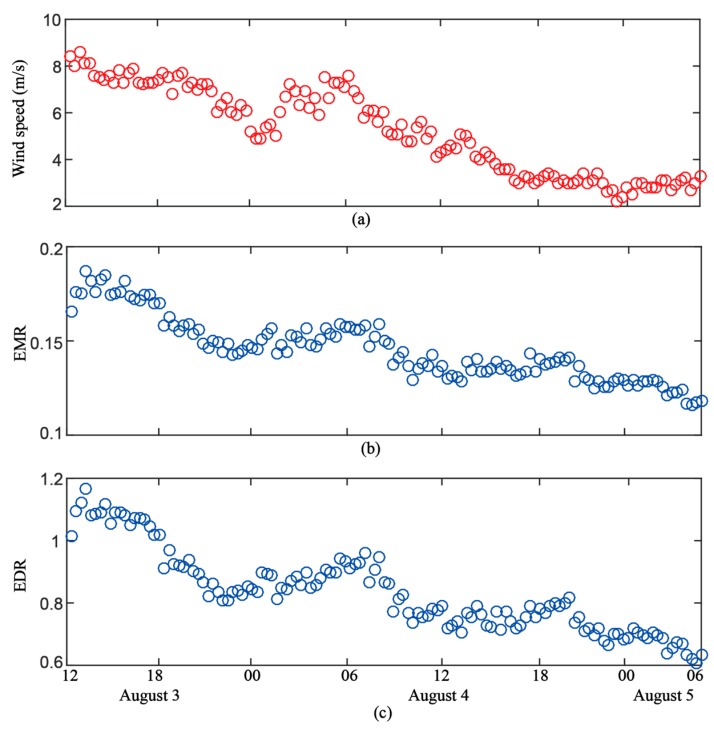
Comparison between in situ wind speed measurement and observables in the gentle wind scenario. (**a**) In situ wind speed. (**b**) EMR. (**c**) EDR. With Threshold2=0.3, both EMR and EDR are computed from the normalized power waveform.

During the coastal experiment, the sea surface wind data from the Zhapo meteorological station was collected every 5 minutes in two periods, i.e. from August 3 to August 5 and from August 13 to August 14. Since these two periods include two typical wind states: gentle wind (1~10 m/s) and high wind (10 m/s~40 m/s), respectively, we chose them to verify the performance of the new observables for wind retrieval. From the afternoon of August 3 to the early morning of August 5, the trend of the wind shows a decreasing evolution ranging from 10 m/s to 2 m/s as shown in Figure 6a. To the contrary, the wind speed shows an increasing evolution from noon on August 13 to the early morning on August 14. The wind speed ranged from 12.7 m/s to 37.3 m/s during the observation time.

### 4.2. Energy Observables Result

#### 4.2.1. Gentle Wind Scenario

The energy observables (EMR and EDR) extracted from August 3 to August 5 are shown in Figure 6b,c with Threshold2=0.3. To study the relationship between the observables and in situ wind speed, we resampled the in situ data to match the temporal resolution of the observables. From Figure 6b,c, both observables show the same evolution with in situ wind speed. 

**Figure 7:**
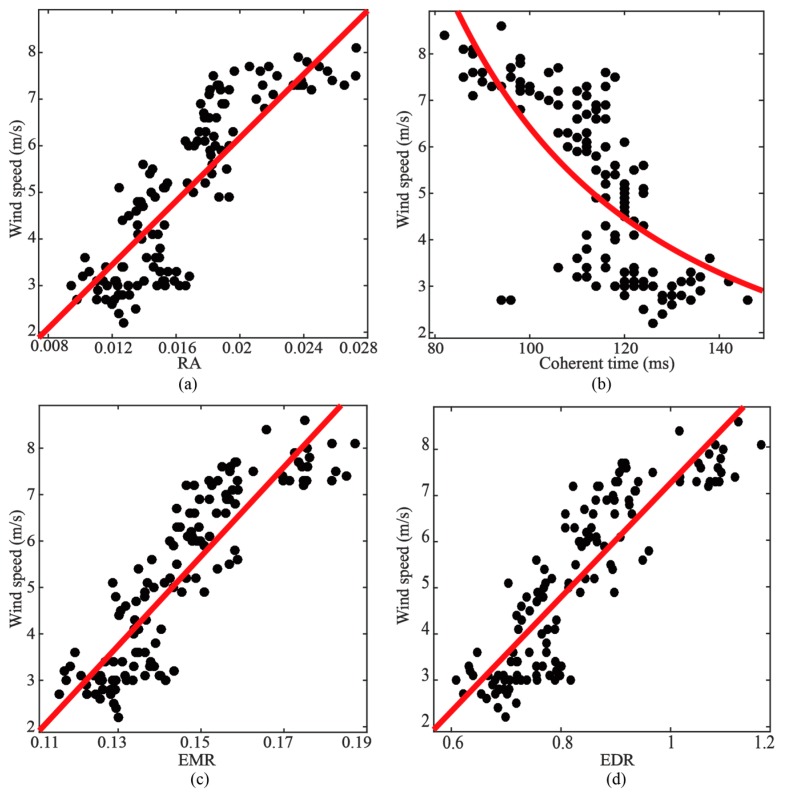
Scatter plots between in situ wind speed and observables in the gentle wind scenario. (**a**) RA. (**b**) Coherent time. (**c**) EMR. (**d**) EDR. EMR and EDR are computed with Threshold2=0.3. The red lines are fitting curves.

The scatter plots between wind speed and EMR are presented in Figure 7c, in which a strong linear dependence on wind speed of EMR can be observed. A simple linear polynomial is employed to describe their relationships.

(11) WS=m⋅EMR+n

where *m* and *n* are coefficients that can be obtained using least squares to fit EMR and in situ wind speed. To evaluate the wind speed retrieval performances, two metrics including the root mean square (RMSE) and the coefficient of determination were computed. As shown in Table 3, the correlation between EMR and wind speed is 0.769, with a RMSE of 0.89 m/s.

As shown in Figure 7d, the scatter plots also show a strong linear relationship between wind speed and EDR. A similar linear polynomial was employed to describe their relationship.

(12) WS=a⋅EDR+b

where a and b are fitted coefficients. The fitting result indicates a correlation of 0.765, with a RMSE of 0.90 m/s.

Two metrics of wind speed retrieval including coherent time and relative amplitude (RA) [18,19] are employed to compare the retrieval performances with the new observables. The coherent time is the time that the autocorrelation of the complex value in specular delay point decays to 1/e of its maximum. The RA is the amplitude ratio of a delay point in the trailing edge and specular point. Figure 7a,b illustrate the relationships between the wind speed, RA and the coherent time, respectively. As presented in Table 3, both methods show good retrieval performances in the gentle wind scenario. 

#### 4.2.2. High Wind Scenario

**Figure 8:**
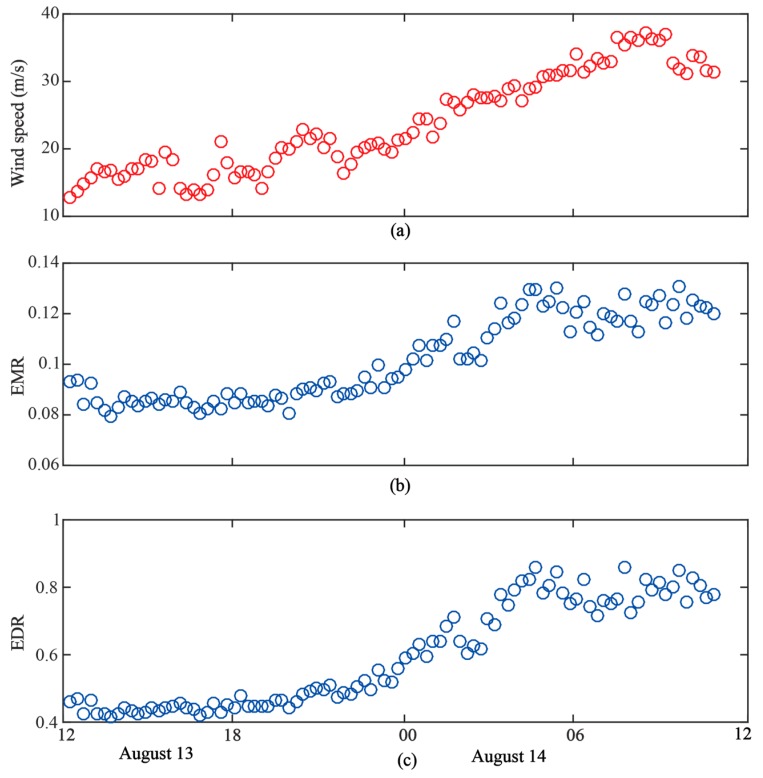
Comparison between in situ wind speed measurement and observables during the Typhoon UTOR. (**a**) In situ wind speed. (**b**) EMR. (**c**) EDR. EMR and EDR are computed with Threshold2=0.25.

In this section, the proposed observables were measured during Typhoon UTOR. UTOR started as a tropical depression and evolved into a typhoon on August 11 in the western north Pacific. As an example, EMR and EDR were computed with the Threshold2 set to 0.25. A detailed discussion on threshold will be presented in Section 4.3. The evolution of EMR and EDR from August 13 to August 14 are presented in Figure 8b,c. Comparing with the in situ wind speed in Figure 8a, both EDR and EMR present the same trends as illustrated in Figure 8b,c. 

**Figure 9:**
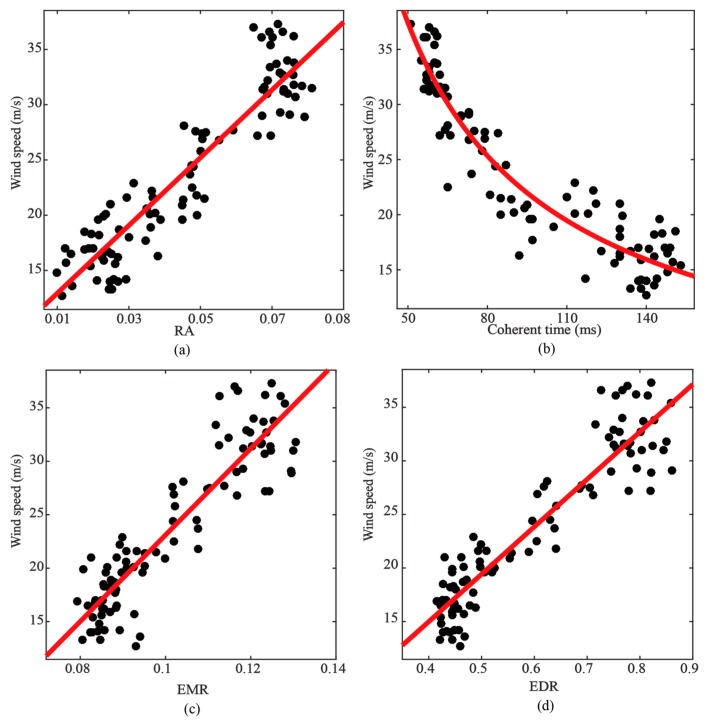
Scatter plots between in situ wind speed and observables during Typhoon UTOR. (**a**) *RA*. (**b**) Coherent time. (**c**) EMR. (**d**) EDR. EMR and EDR are computed with Threshold2=0.25. The red lines are the fitting curves.

The scatter plots between the wind speed and EMR and EDR during Typhoon UTOR are presented in Figure 9c,d, respectively. Both EMR and EDR show an obvious linear dependence on wind speed. A simple linear polynomial is employed to describe the relationship between wind speed and EMR.

(13) WS=p⋅EMR+q

where p and q are fitting coefficients. As shown in Table 4, the correlation between EMR and wind speed is 0.822, with a RMSE of 3.10 m/s. A similar linear polynomial was employed to establish the relationship between wind speed and EDR.

(14) WS=k⋅EDR+c

where k and c are fitting coefficients. The fitting result with a correlation of 0.866 and a RMSE of 2.69 m/s was obtained.

As shown in Figure 9a,b, both coherent time and the RA method were also used for wind speed retrieval. Comparing the results for the coherent time and RA methods, the coherent time shows a slightly better retrieval performance when employing a non-linear fitting, with a correlation coefficient of 0.875 and a RMSE of 2.61 m/s.

### 4.3. Discusson

#### 4.3.1. Threshold Effects

This paper defined two energy zones in the trailing edge of the normalized power waveform by setting two thresholds as

(15) $$\left\{ \begin{array}{l} Threshold1=P_{noise}\\ Threshold1<Threshold2<1 \end{array}\right.$$

where $P_{noise}$ is the noise power, which can be estimated by averaging the samples of the waveform with no scattered signals. Specifically, we used delay values between −2 and −1.5 chips before the specular point to estimate the noise. Since Threshold2 is an important factor when defining the energy zones, this section compared different values of Threshold2 in order to find the optimal threshold for wind speed retrieval. Table 5 shows the wind speed retrieval performances under different threshold values.

**Figure 10:**
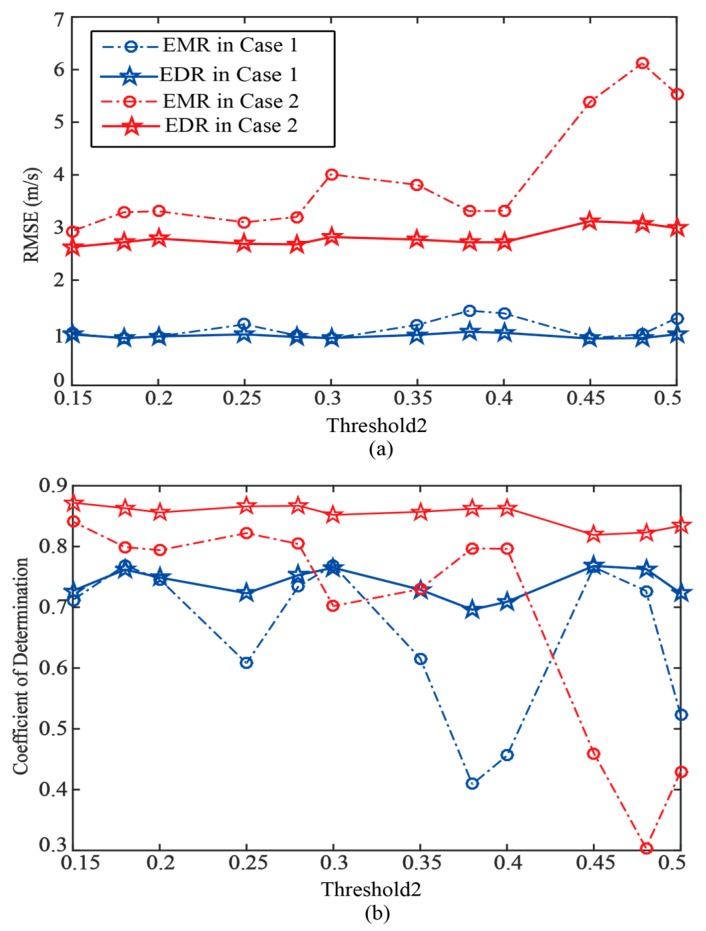
(**a**) RMSE and (**b**) coefficient of determination $R^{2}$ as a function of values of Threshold2. Case 1 is in the gentle wind scenario, Case 2 is in the UTOR scenario.

As presented in Table 5, the EMR method shows a fluctuant correlation level with respect to different Threshold2 in both scenarios. Alternatively, the EDR method presents a relatively stable correlation value of around 0.73 and 0.85 in two wind scenarios, respectively. Figure 10 illustrates the wind speed retrieval performances with different Threshold2 ranging from 0.15 to 0.5. In the gentle wind scenario (Case1, the blue line), the EMR reaches a relatively better correlation of 0.769 when Threshold2 is 0.3 and degrades rapidly when Threshold2 is increasing from 0.3 to 0.38. In the high wind scenario (Case 2, the red line), the EMR correlation level shows a considerable fluctuation when Threshold2 ranges from 0.28 to 0.25. The EDR keeps a stable retrieval performance in both scenarios. 

As shown in Figure 10, in the gentle wind scenario, when Threshold2 is 0.3, the wind speed retrieval RMSE by EMR and EDR methods are 0.89 m/s and 0.90 m/s and $R^{2}$ are 0.769 and 0.765, respectively. In the high wind scenario, when Threshold2 is 0.15, the RMSE by EMR and EDR methods are 2.93 m/s and 2.63 m/s and $R^{2}$ are 0.841 and 0.871. According to these results, the optimal value of Threshold2 for wind speed retrieval is 0.3 in the gentle and high wind scenarios. In the high wind scenario, the optimal Threshold2 is 0.15. 

#### 4.3.2. Coherent Integration Time Effects

**Figure 11:**
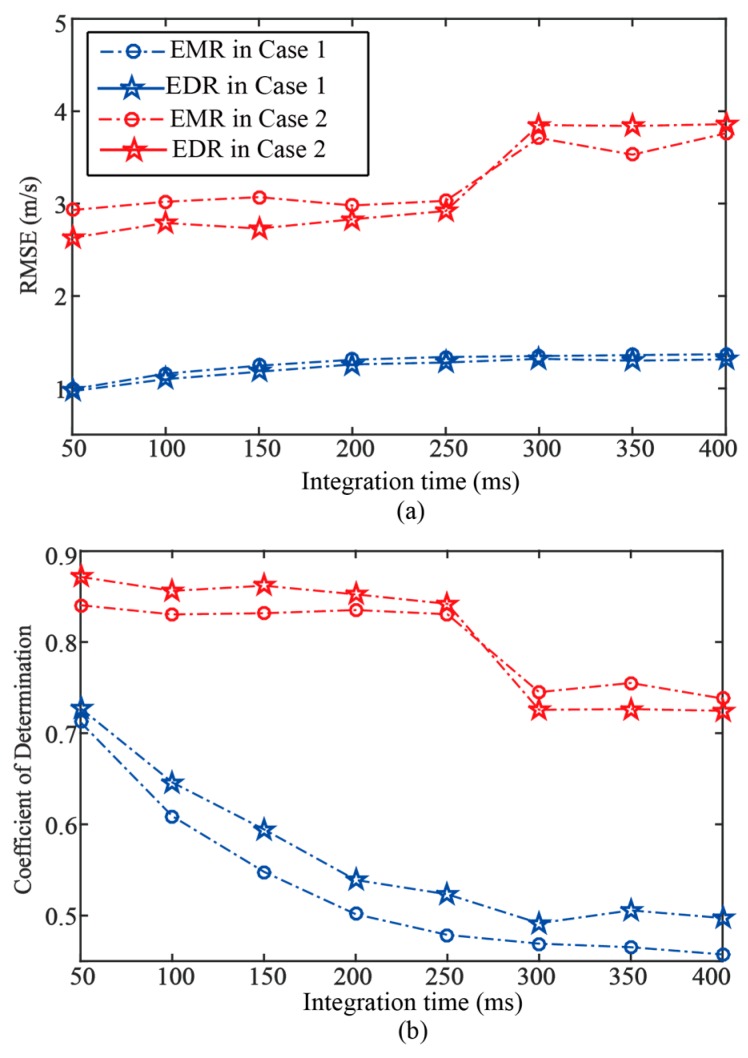
(**a**) RMSE and (**b**) coefficient of determination $R^{2}$ as a function of the coherent integration time. Case1 is the gentle wind scenario, Case 2 is in the UTOR scenario.

As the scattered signals are contaminated by additive thermal noise and speckle noise, the coherent integration was employed over the complex waveforms to suppress the effect. We tested different lengths of coherent integration time to study their effect on wind speed retrieval and to achieve an optimum value of coherent integration time. Figure 11 presents the results for different coherent integration time. Both methods show degradations in the wind speed retrieval performances with increasing coherent integration time. 

In order to keep the correlation of the waveform, the coherent integration time should be smaller than the coherent time of the scattered signal [18]. In our coastal experiment scenario, the coherent time ranged from 50 ms to 150 ms, depending on the sea state. Therefore, the best performances were obtained as expected for a coherent integration time of 50 ms, in agreement with the principle that the coherent integration time should be smaller than the correlation time.

## 5. Conclusions

Two new observables that describe the energy distribution of delay waveform were proposed in this paper. To study the dependence of the reflected GNSS signal on the sea surface wind speed, we divided the complex waveform into two zones, i.e. low-energy zone and high-energy zone, and proposed two new observables as input for alternative methods for wind speed retrieval. EMR is the ratio of energy between the low-energy zone and the high-energy zone. EDR is based on the computation of the ratio of the pixel volume between the low-energy zone and the high-energy zone. This paper processed two periods of dataset from the Beidou GEO satellite during the coastal experiment in Yangjiang, which represented two typical different wind states: gentle and high wind scenarios. Both observables showed good wind speed retrieval performances in the gentle wind scenario. The wind speed retrieval errors were less than 1.0 m/s with correlation $R^{2}$ of 0.76 when Threshold2=0.3. In the high wind scenario, the dataset during Typhoon UTOR were processed. When Threshold2=0.25, EMR and EDR showed a strong linear relationship with the high wind speed during Typhoon UTOR, which reached a RMSE of 3.10 m/s and 2.69 m/s with a correlation $R^{2}$ of 0.822 and 0.866, respectively. 

To obtain an optimal retrieval result, the influences of threshold and coherent time on wind speed retrieval were analyzed. Finally, the optimal retrieval performances were obtained, with an RMSE of 2.63 m/s and a correlation of 0.871 in the high wind scenario. In the gentle wind scenario, the optimal RMSE of wind speed reached 0.89 m/s and the correlation was 0.769.

We neglected the wind direction factor when we set up the wind speed retrieval models in this paper. In the future, the relationships between wind direction and delay waveform and influences of wind direction on waveform will be considered, which will be good for estimating a more efficient wind speed retrieval model. Besides, it should be remarked that the buoy is located near the gulf of the Yangjiang Island and the distance between the GNSS coastal experiment station and the in situ measurement station is around 10 km, which may introduce additional errors when fitting the retrieval model. By referring to more ocean meteorological information such as tide and swell during the experiment, this may provide more opportunity to investigate the relationship between wind speed and observables in the future.

## Figures and Tables

**Table 1:** Simulation Parameters.

Parameters	Value	Unit
Antenna Gain	12	dB
Antenna beam width	38	deg
Satellite altitude	20200	km
Receiver altitude	120	m
Elevation	31	deg
Frequency	1561.098	MHz
Delay range	−1~2	chip
Wind speed	2, 6, 10, 20	m/s
Wind direction	20, 40, 60, 80	deg

**Table 2:** Configuration of Global Navigation Satellite System (GNSS) raw signal collector.

Parameter	Value	Unit
Radio frequency (RF) Frequency	1561.098	MHz
Intermediate frequency (IF) Frequency	3.996	MHz
Bandwidth	4	MHz
Sample Rate	16.369	MHz
Quantization	2	bit

**Table 3:** Retrieval performances by different observables in gentle wind scenario.

Observable	$R^{2}$	Root Mean Square Error (RMSE) (m/s)
Relative amplitude (RA)	0.744	0.94
EDR	0.765	0.90
EMR	0.769	0.89
Coherent time	0.713	0.98

**Table 4:** Retrieval performances by different observables during Typhoon UTOR.

Observable	$R^{2}$	RMSE (m/s)
RA	0.852	2.82
EDR	0.866	2.69
EMR	0.822	3.10
Coherent time	0.875	2.61

**Table 5:** Wind speed retrieval performances under different threshold values. Case 1: In the gentle wind scenario. Case 2: In the high wind scenario.

Threshold2	0.5	0.4	0.3	0.25	0.2	0.15
***R*^2^ and RMSE (m/s) in Case 1**						
EMR	0.523/1.28	0.456/1.37	0.769/0.89	0.608/1.16	0.746/0.93	0.710/0.98
EDR	0.723/0.97	0.709/1.0	0.765/0.90	0.723/0.97	0.749/0.93	0.718/0.98
***R*^2^ and RMSE (m/s) in Case 2**						
EMR	0.430/5.54	0.796/3.32	0.702/4.01	0.822/3.10	0.794/3.31	2.93/0.841
EDR	0.834/2.99	0.863/2.72	0.852/2.82	0.866/2.69	0.856/2.79	2.63/0.871
